# Assessing the Physiology and Biochemistry of Freshwater Microalgae for Biotechnological Applications

**DOI:** 10.1007/s12010-025-05578-6

**Published:** 2026-02-02

**Authors:** Lucas S. Solidade, Leonardo de Faria, Ana T. Lombardi

**Affiliations:** 1 Biotechnology Graduate Program, Federal University of São Carlos, São Carlos, São Paulo, Brazil; 2 Botany Department, Federal University of São Carlos, São Carlos, São Paulo, Brazil

**Keywords:** Biomolecules, Nutraceutical, Biodiesel, Algae physiology

## Abstract

Microalgae have gained prominence in the food, feed, and bioenergy sectors due to their capacity to produce valuable molecules and mitigate CO_2_, but poor knowledge on species composition limits their applications. This study addresses the physiology of 12 freshwater microalgae belonging to Chlorophyta (*Chlorella emersonii*,* Desmodesmus brasiliensis*,* Dimorphococcus* sp., *Pediastrum* sp., *Raphidocelis* sp., *Radiococcus* sp., *Tetranephris brasiliensis*,* Westella botryoides*), Charophyta (*Staurastrum leptocladum*,* Staurastrum pantanale)*, Cryptista* (Cryptomonas obovata)*,* and Heterokontophyta (Ophiocytium* sp.). Physiological parameters as light saturation index (E_k_), growth rate, dry biomass (DW) yield and the biochemical composition of the cells were determined. The results showed that E_k_ varied within 154 (*T. brasiliensis*) and 312 (*C. emersonii)* µmol photons.m^− 2^.s^− 1^. *Pediastrum* sp. and *C. emersonii* presented the highest growth rate (~ 1.0 d^− 1^), while *S. leptocladum* the best antioxidant activity (43% DPPH inhibition). The Chlorophyta had the highest protein content (60% DW), while highest carbohydrate (40% DW) were present in the Charophyta. *Dimorphococcus* sp. and *S. pantanale* stood out for total lipids (25–30% DW). *W. botryoides* had 80% of its fatty acids as polyunsaturated molecules, dominated by alpha-linolenic acid and linoleic acid, followed by *C. obovata*, *Pediastrum* sp., *S. leptocladum*, and *S. pantanale* with 60%. Highest saturated and monounsaturated fatty acids (~ 28%) were present in *Dimorphococcus* sp. We highlight *Pediastrum* sp. for food applications because of its total proteins and polyunsaturated fatty acids content, and *Dimorphococcus* sp. for biofuels applications due to the saturated lipids. This work expands the knowledge on underexplored microalgae for biotechnological applications.

**Supplementary Information:**

The online version contains supplementary material available at 10.1007/s12010-025-05578-6.

## Introduction

Microalgae are photosynthetic organisms with positive impact on reducing atmospheric CO_2_, an important greenhouse gas [1]. Microalgae have physiological plasticity, and their biochemical composition may change as growth conditions varies [2], thus, to investigate their physiology, culture conditions should be precisely defined. Light intensity required for optimal growth is a fundamental aspect to be thought of when aiming at productive algal cultures. Additionally, to be able to compare different species, it is important that cultures be sampled in exponential growth, when the cells reveal their optimum metabolism. This is because, in batch cultures, cells experience changing environmental conditions over time, making it difficult to determine the exact factors influencing their growth and photosynthesis [3]. For ease of comparison with existing literature and among species, in the present study, cells were harvested in the exponential growth phase, with each species cultured under its respective E_k_.

Microalgae synthesize a diverse range of biomolecules such as proteins, carbohydrates, and high value-added lipids, some of them produced exclusively by microalgae. Compared to terrestrial crops, microalgae can achieve significantly higher biomass productivity per hectare, producing up to ten times more proteins, carbohydrates, and lipids than soybean [4]. The possibility of using microalgae in several industrial sectors has led to significant interest in their production [5–7], but so far, their biotechnological use is limited, and industrial scale applies to just few species.

Among the most extensively studied species, *Chlorella vulgaris* and *Limnospira platensis*, stand out as protein producers. They are commercialized as food supplement, the first a Chlorophyta (50–60% proteins) and the second a Cyanobacteria (60–70% proteins). Each one has its own advantage, for example *Chlorella* accumulates unsaturated fatty acids in addition to proteins, but contains cellulose in its cell wall, whereas the Cyanobacteria has low lipid content and lack cellulose, making them easier to digest [8]. Other species, such as *Botryococcus braunii*, are recognized for their lipid accumulation, reaching up to 50% of their dry biomass under optimal conditions and, in some cases, increasing to 70% under stress [9, 10]. Some of these lipids have commercial relevance, such as palmitic and oleic acids for biofuel production, however, there is no microalgae commercial plant for their production yet. Among the lipids, omega-3 fatty acids are essential for human health and have important nutritional value. These biomolecules position microalgae as promising candidates for numerous biotechnological applications, including functional food ingredients and sustainable energy products.

Despite the growing interest in microalgae, commercial production remains limited to a few genera, including *Limnospira*, *Chlorella*, *Dunaliella*, *Aphanizomenon*, and *Haematococcus* [11]. This restriction can be related to factors such as regulatory barriers, insufficient biochemical characterization, and challenges in optimizing large-scale cultivation conditions, all of which impact economic feasibility. Currently, only a limited number of species are approved for human consumption, e.g., eight in the USA, thirteen in the European Union, and seven in China [12]. Considering the vast number of estimated species (180,199 - https://www.algaebase.org/), expanding our knowledge and physiological characteristics of microalgae species could reveal new strains with valuable industrial potential.

This study investigated the physiology of twelve freshwater microalgae species under controlled laboratory conditions, evaluating their biochemical composition and assessing their potential as sources of proteins, carbohydrates, lipids, fatty acids, and antioxidant compounds. By identifying promising strains based on growth rate and biochemical composition, we selected those with potential to be applied in different industrial sectors. This research contributes to broadening the range of commercially valuable microalgae, therefore expanding their applications in biotechnology.

## Materials and methods

### Cultures

The strains investigated were obtained from the Freshwater microalgae culture collection of the Botany Department, Federal University of São Carlos (CCMA-UFSCar) and the Freshwater Microalgae Culture Collection of the Institute of Biological Sciences at the Federal University of Rio Grande (FURG). In the culture collections, the algae are kept in WC medium [13]. However, for the experiments, they were adapted to the BG11 medium [14] because it is a nutrient richer medium that supports higher biomass. Those that did not adapt to BG11 were maintained in WC. Before the experiments, the minimum saturating light intensity (E_k_) for each strain was determined through rapid light curves using a PhytoPAM (Heinz-Walz Effeltrich, Germany) instrument. The species used in the present investigation are presented in Table 1 with their respective code from the culture collection and the taxonomic Phylum.

**Table 1:** Microalgae species used in the present investigation, with their respective culture collection identification code and taxonomic phylum

Code	Microalgae	Phylum
CCMA323	*Chlorella emersonii Shihira & R.W.Krauss*	Chlorophyta
CCMA148	*Cryptomonas obovata* Skuja	Cryptista
CCMA262	*Desmodesmus brasiliensis* (Bohlin) E.Hegewald 2000	Chlorophyta
CCMA571	*Dimorphococcus* sp. Braun	Chlorophyta
CCMA553	*Ophiocytium* sp. Nägeli	Heterokontophyta
CH007	*Pediastrum* sp. Meyen	Chlorophyta
CCMA655	*Radiococcus* sp. Schmidle	Chlorophyta
CCMA498	*Raphidocelis sp.* Hindák	Chlorophyta
CCMA200	*Staurastrum leptocladum* Nordstedt	Charophyta
CCMA382	*Staurastrum pantanale* K.R.de Souza Santos, C.F. da Silva Malone, C.Leite Sant’Anna & C.E.M.Bicudo	Charophyta
CCMA307	*Tetranephris brasiliensis* Leite & C.E.M.Bicudo	Chlorophyta
CCMA311	*Westella botryoides* (West) De Wildeman	Chlorophyta

Microalgae were grown in batch cultures performed in 1 L Houx-type flasks (267 mm x 122 mm x 56 mm) with 800 mL of the appropriate culture medium for each species. The cells were exposed to the respective species E_k_, with constant aeration of filtered air (0.22 μm) and a 12 h/12 h light/dark photoperiod and controlled temperature (25 °C *±* 1). The microalgae were grown up to the end of the exponential growth phase, when they were harvested. Culture monitoring was performed daily for population growth by in vivo chlorophyll a fluorescence (Turner fluorometer - USA) and absorption at 684 nm in a spectrophotometer (NANOCOLOR^®^ UV/VIS - MACHEREY-NAGEL, Germany).

The specific growth rate (µ) was calculated based on a linear regression fit of the fluorescence data by plotting the natural logarithm of in vivo fluorescence (Y axis) against cultivation time (X axis). The slope of the straight line represents the specific growth rate in the exponential growth phase. Three experimental replicates were performed.

### Dry Biomass and Biochemical Composition

Dry biomass was determined by filtering 5 mL of culture through a 0.22 μm cellulose acetate filter (Whatman, England) of previously determined mass using a 10^− 6^ g precision balance (Mettler Toledo XPE26, Switzerland). The filters were then dried in an oven (40 °C) until constant mass and weighted using the same balance. At the end of the culture, its total volume was centrifuged (Sorvall Legend XTR, Thermo Fisher Scientific, Osterode, Germany) and lyophilized, and the dry biomass was kept frozen (−80 °C, Haier Biomedical, DW-88–486, Qingdao, China) until use. Biomass productivity was calculated by dividing the final biomass concentration by the number of cultivation days considered for the culture harvesting.

Culture sampling for biomolecules and pigments was performed in exponentially growing cells. As this study investigates different microalgae strains, examining them during the exponential growth phase can ensure a consistent comparison among them, since the reasons for a batch culture entering stationary phase may vary from species to species. Total proteins content was determined according to Slocombe et al. [15] and the biomass digested as in Price [16]. The protocol is based on proteins quantification with Folin reagent adapted for microplates, and the absorbance was determined in a microplate spectrophotometer (Epoch - Biotech, USA). Total carbohydrates concentration was determined in cells previously digested with sulfuric acid and absorbance quantified in 315 nm. The calibration curve was performed with glucose as standard [17]. Total lipids content was determined by gravimetry according to Parrish [18], a modification of the Folch et al. method [19]. The results obtained are expressed as the percentage of biomolecules in relation to dry biomass weight (% DW).

The photosynthetic pigments chlorophyll (Chl) a and b, and total carotenoids were determined as described in Shoaf and Lium [20] and Wellburn [21] for the Chlorophyta and Charophyta. For the Heterokontophyta, Xanthophyceae (*Ophiocytium* sp.), which have Chl a, c1 and c2, but not Chl b, and the Cryptista that have Chl a and c2, the photosynthetic pigments were determined according to Ritchie et al. [22]. For this analysis 3 mL culture samples were filtered through a 0.22 μm cellulose acetate filter (Whatman, England) and the filter containing the biomass was used for the pigment extraction with dimethyl sulfoxide and spectrophotometric determination. Absorbance was measured using a NANOCOLOR^®^ UV/VIS (MACHEREY-NAGEL, Germany) spectrophotometer. The results are presented in mg.g^− 1^ of dry biomass.

### Rapid Light Curves (RLC)

A pulse amplitude modulation fluorometer (Phyto-PAM, Walz – Germany) was used for performance of the rapid light curves using the instrument settings. For measurements, cultures were harvested in exponential growth phase, and the samples were dark adapted for 20 min. After this, they were exposed to photosynthetically active radiation (PAR) of increasing intensity, from 0 to 1800 µmol photons m⁻²s⁻¹ every 20 s. The relative electron transport rate (rETR, µmol electrons m^− 2^ .s^− 1^) was calculated by multiplying the PAR intensity by the respective effective quantum yield and plotted against PAR intensity. The curve was fitted using the mathematical model described in Platt et al. [23] and the rETRmax and α obtained. From these, the E_k_ value was calculated as rETRmax/α (µmol photons m⁻².s⁻¹). The calculations and data plot were performed using the R Package Phytotools and ggplot2.

### Fatty Acids

Fatty acids profile was determined after the direct transesterification methodology proposed by Soares et al. [24] onto dry biomass. The preparation of fatty acid methyl esters (FAMEs) was performed using lyophilized biomass (Solab© SL − 404). The methyl esters were collected in vials and dried under a nitrogen stream and solubilized in heptane 99% (Exodo Científica, Brazil) for analysis. An Agilent 7890 gas chromatograph coupled to an Agilent 5975 mass spectrometer (Agilent Technologies, Inc. USA) with an Agilent HP-5MS column (5% Phenyl Methyl Silox 30 m x 250 μm x 0.25 μm) was used to analyze the FAMEs. The chromatographic analysis was conducted with 70 eV electron impact energy as the electron energy, ion source temperature was 230 °C, and transfer line temperature 280 °C. The injection volume was 1 µL using a micro syringe (Agilent - AG5181-1267) in splitless mode with an injector temperature of 120 °C. Helium 5.0 (> 99.999% pure) was used as the carrier gas at a pressure of 15,926 psi in flow of 1.924 mL.min^− 1^. The initial temperature was 70 °C for the first 10 min and heated at a constant rate of 5 °C.min^− 1^ until 250 °C, remaining constant thereafter up to the end of the run. The FAMEs identification was based on a FAME MIX C8-C24 standard (Supelco Product number 18918) and using the NIST11.L library (National Institute of Standards and Technology). AMDIS 32 Analysis software (Automated Mass Spectral Deconvolution & Identification System) associated with the mass spectra files present in the AOCS “Lipid library” of the Scottish Crop Science Research Institute (http://www.lipidlibrary.com.uk/índex.html) was used. Fatty acid normalization was based on integrated peak area.

### Antioxidant Activity and Total Polyphenols

The antioxidant activity was determined in methanolic extracts prepared using lyophilized microalgae biomass and HPLC grade methanol (J.T. Baker, UK) at a ratio of 50 mg of biomass per mL of methanol. After adding the solvent, the solution was vortexed (Gehaka© AV-2) for 1 min with glass beads (3 mm diameter) to facilitate cell rupture. After the extraction step, the samples were centrifuged in a refrigerated centrifuge at 2486 g and 10 °C (Thermo Scientific© Sorvall ST16-R) for 15 min. The extraction was repeated three times, and the supernatants were collected and used for antioxidant activity and total polyphenols determinations. The antioxidant activity was assessed using the DPPH (2,2-diphenyl-1-picrylhydrazyl) radical reduction method, adapted for microplates [25]. The calculation was made according to Eq. 1.

1 $$\:DPPH\:Scavenging\:activity\:\left(\%\right)=\:\frac{\left({Abs}_{control}-\:{Abs}_{sample}\right)}{{Abs}_{control}}\:.\:\:100$$

Total polyphenols assay was performed using the Folin-Ciocalteu method adapted for microplates [26]. The analysis was performed with extracts at 1:10 ratio using 60 µL of sample, followed by 60 µL of 1:1 Folin-Ciocalteu reagent (Sigma-Aldrich, 2 N, Switzerland) and 80 µL of 7.5% sodium carbonate solution. The solution was incubated at room temperature for 2 h and then absorbance was determined at 750 nm in a spectrophotometer (NANOCOLOR^®^ UV/VIS - MACHEREY-NAGEL, Germany). For the blanks, only microalgae extracts at 1:10 ratio were used with the same final volume as the quantified samples. The values were calculated according to a calibration curve made with gallic acid and expressed in gallic acid equivalents per gram of biomass (mgEq.g^− 1^).

#### Statistical Analysis

Statistical analyses were performed using one-way analysis of variance (ANOVA) and compared using Tukey’s test with a significance level of 95%. The Shapiro-Wilk tests for normality and Levene’s test for homogeneity of variances were performed. RStudio version 2023.03.0, with R 4.2.2 and the stats and agricolae packages were used for statistics, ggplot2 for graphics, Factoshiny and missMDA for the principal component analysis.

## Results

### Growth rates, Biomass and Productivity

**Fig. 1:**
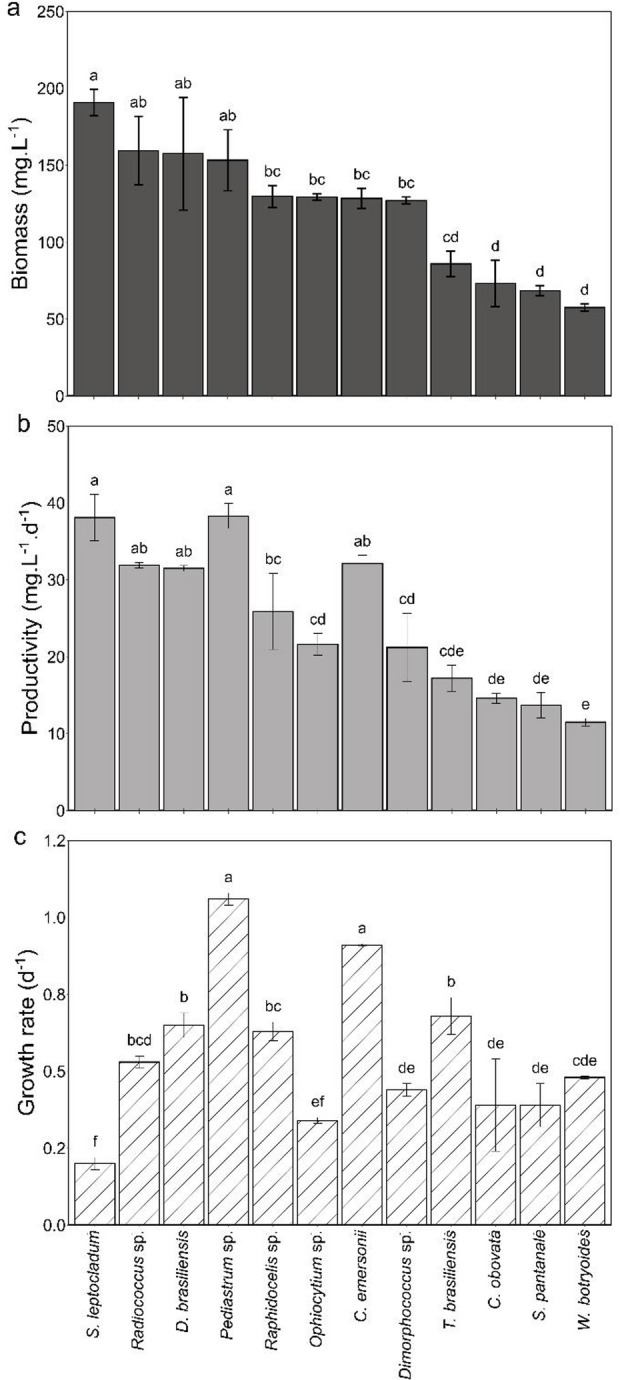
Production parameters for the twelve microalgae species studied in the present research ordered by biomass. In (**a**) Final biomass (mg.L⁻¹), (**b**) biomass productivity (mg.L⁻¹.d⁻¹), and (**c**) growth rate (**d**⁻¹) based on in vivo chlorophyll a fluorescence. Values represent the mean (*n* = 3) and error bars the standard deviation of the mean (*n* = 3). Same letters indicate no statistically significant difference (*p-value* < 0.05)

The exponential growth phase of the microalgae ranged from 4 to 6 days depending on the species. Figure 1 shows specific biomass, productivity and growth rates obtained for the cultures.

Growth rates ranged from 0.20 ± 0.02 d⁻¹ to 1.06 ± 0.02 d⁻¹, with *Pediastrum* sp. and *C. emersonii* showing the highest values (statistically similar values), and *S. leptocladum* the lowest (0.20 ± 0.02 d⁻¹). Despite its low growth rate, cultures of *S. leptocladum* achieved the highest biomass concentration (190.7 ± 8.50 mg.L⁻¹). Similarly, *Radiococcus* sp. and *Desmodesmus brasiliensis* showed high biomass values (159.6 ± 22.3 mg.L⁻¹ and 157.6 ± 36.6 mg.L⁻¹, respectively), but moderate growth rates. Biomass productivity was highest in *Pediastrum* sp. (38.33 ± 1.62 mg.L⁻¹.d⁻¹) and *S. leptocladum* (38.14 ± 3.01 mg.L⁻¹.d⁻¹). In contrast, *W. botryoides* exhibited the lowest productivity (11.49 ± 0.49 mg.L⁻¹.d⁻¹), associated with low biomass production.

### Rapid Light Curves

**Fig. 2:**
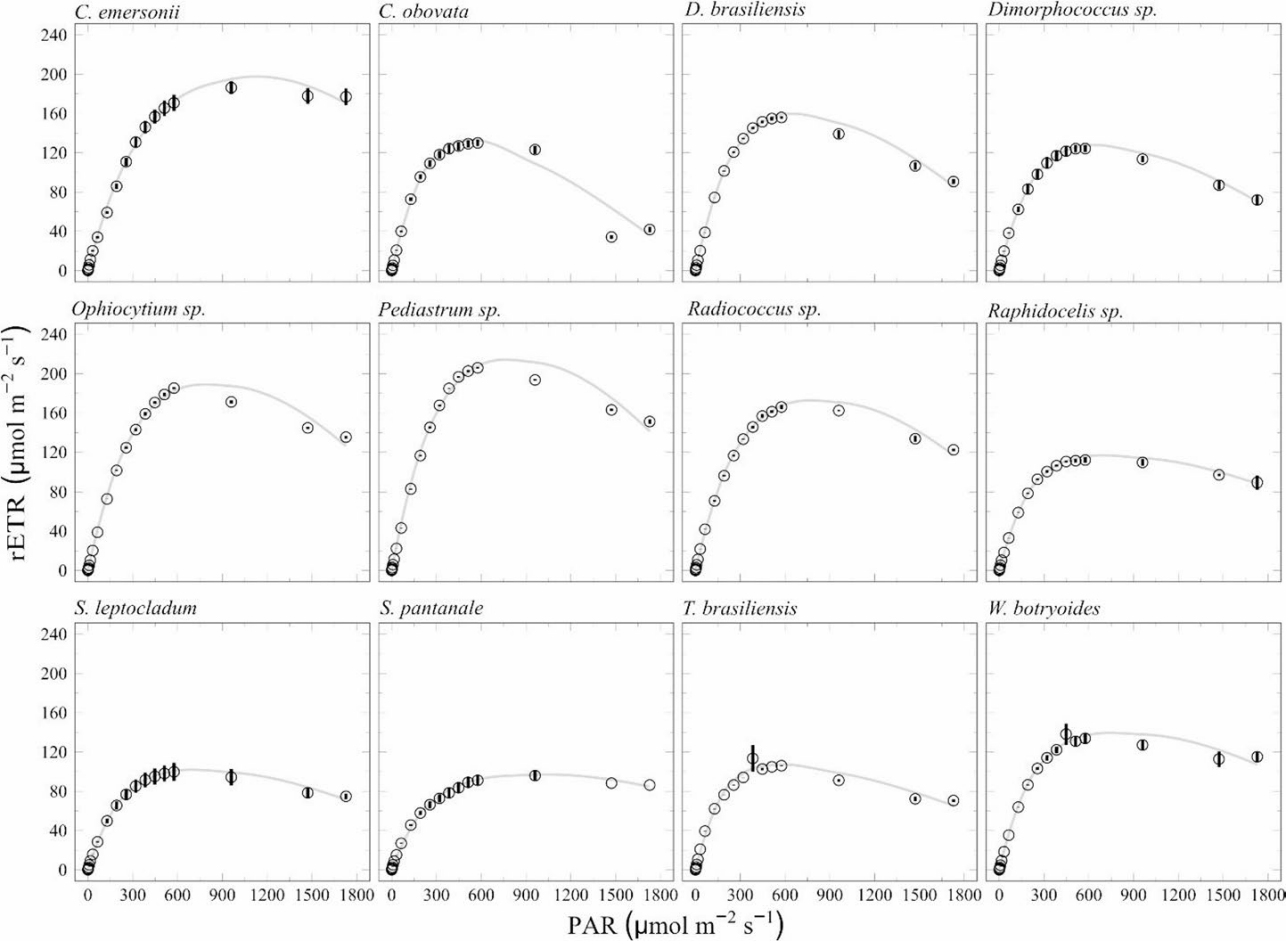
Rapid Light Curves obtained for the microalgae, fitted according to Platt et al. (1981). Y axis represents the relative electron transport rate in µmol electrons.m^−2^.s^−1^ and X axis represents the photosynthetic active irradiance (PAR) in µmol photons.m^−2^.s^−1^ Data points represent the means and error bars the standard deviation (*n* = 3)

Figure 2 shows the rapid light curves (RLC) obtained for the 12 microalgae and the parameters a (a representation of light efficiency utilization), and rETRmax (relative maximum electron transport rate) obtained after the curve fitting. The saturating irradiance (E_k_) values, an important parameter for microalgae growth and physiology, are reported in Table 2.

**Table 2:** Rapid light curve parameters obtained for the microalgae. Alpha (α): light-harvesting efficiency, rETR_max_: maximum relative electron transport rate (µmol e.m^−2^.s^−1^), and ek: saturating irradiance (µmol photons.m^−2^.s^−1^). Same letters represent no statistical difference (ANOVA, *p-value* < 0.05). Values represent the mean (*n* = 3), and the standard deviation of the mean is shown in parentheses

Microalgae	Alpha (α)	rETR_max_	Ek
*C. emersonii*	0.61 (0.04)^de^	189.83 (14.84)^ab^	312.02 (3.13)^a^
*C. obovata*	0.72 (0.02)^bc^	131.17 (7.17)^e^	182.49 (1.8)^de^
*D. brasiliensis*	0.75 (0.01)^b^	157.54 (3.12)^cd^	210.8 (4.06)^bcd^
*Dimorphococcus* sp.	0.64 (0.06)^cd^	125.24 (7.14)^ef^	196.79 (10.04)^de^
*Ophiocytium* sp.	0.73 (0.01)^bc^	184.92 (2.90)^ab^	254.18 (0.95)^b^
*Pediastrum* sp.	0.85 (0.01)^a^	209.52 (0.77)^a^	246.18 (4.35)^b^
*Radiococcus* sp.	0.69 (0.02)^bcd^	168.84 (3.29)^bc^	243.29 (8.12)^bc^
*Raphidocelis* sp.	0.68 (0.01)^bcd^	122.40 (11.09)^cde^	180.00 (11.09)^de^
*S. leptocladum.*	0.52 (0.05)^ef^	99.68 (16.13)^g^	192.96 (30.85)^de^
*S. pantanale*	0.44 (0.01)^f^	94.10 (8.04)^g^	213.42 (10.22)^bcd^
*T. brasiliensis*	0.68 (0.01)^bcd^	105.27 (3.49)^fg^	154.65 (4.04)^e^
*W. botryoides*	0.68 (0.01)^bcd^	136.02 (8.99)^de^	200.13 (10.21)^cd^

The light-harvesting efficiency (α) of the examined microalgae species varied significantly. *Pediastrum* sp. showed the highest value, with an α = 0.85, while *S. pantanale* had the lowest, 0.44. Among the algae, *Pediastrum* sp. had the highest rETRmax (209.52 µmol e.m^− 2^.s^− 1^) followed by *C. emersonii* (189.83 µmol e.m^− 2^.s^− 1^) and *Ophiocytium* sp. (184.92 µmol e.m^− 2^.s^− 1^). The lowest rETRmax values were present in *S. pantanale* (94.10 µmol e.m^− 2^.s^− 1^) and *S. leptocladum* (99.68 µmol e.m^− 2^.s^− 1^). For the light saturation parameter (Ek), *C. emersonii* had the highest tolerance to high irradiance, 312.02 µmol photons.m^− 2^.s^− 1^, while *T. brasiliensis* had the lowest (154.65 µmol photons.m^− 2^.s^− 1^). These findings indicate the different photosynthetic capacities, notably pointing out the efficiency in light use of *Pediastrum* sp. and *C. emersonii*.

### Biochemical Composition

**Fig. 3:**
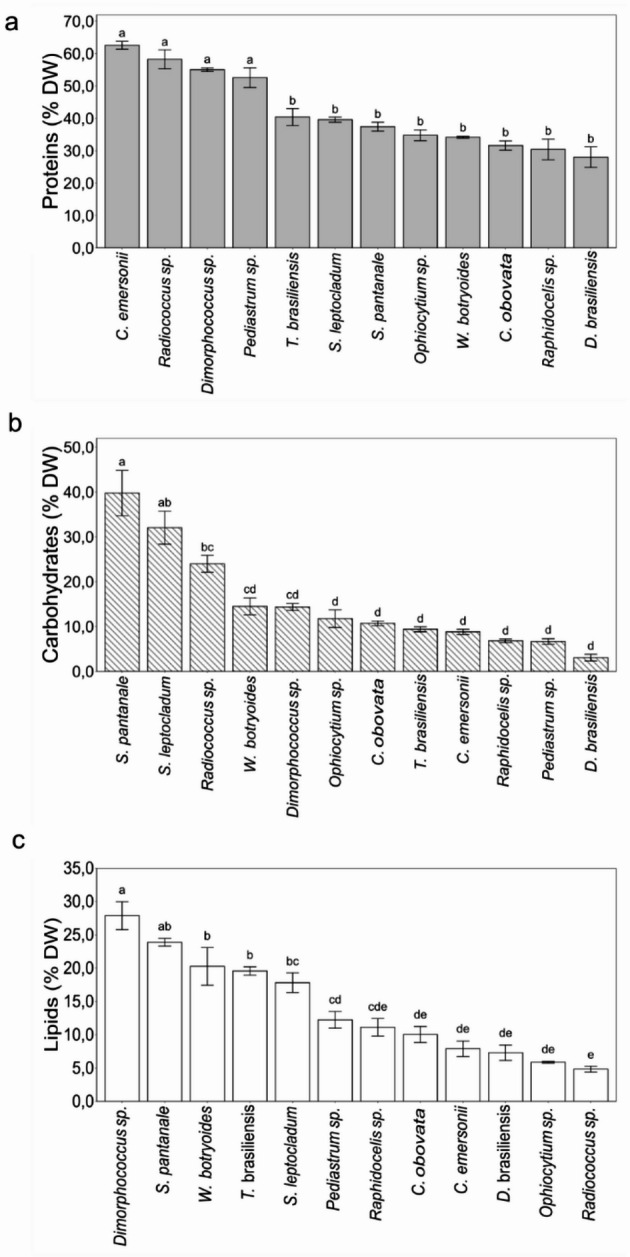
Biochemical composition of the microalgae in percentage of dry biomass (DW). A: total proteins, B: total carbohydrates, C: total lipids. Same letters represent no statistical difference (ANOVA, *p-value* < 0.05). Error bars represent the standard deviation of the mean (*n* = 3)

In this study, some species produced a high content of total proteins, as shown in Fig. 3. *C. emersonii* (63% DW), *Radiococcus* sp. (58% DW), *Dimorphococcus* sp. (56% DW), and *Pediastrum* sp. (52% DW) were the species that produced significant amounts of proteins, with over 50% of their dry biomass. However, in the other species, the total proteins ranged from 30 to 40%.

The carbohydrate was highest in *S. pantanale* (40% DW) as shown in Fig. 3B, a value almost equivalent to that of its total proteins. Despite the slightly lower average, *S. leptocladum* presented statistically equivalent amounts of carbohydrates. *Radiococcus* sp. stood out with 24% DW carbohydrates, while in the other microalgae the biomolecule ranged from 5% to 15% DW. Regarding the total lipid content (Fig. 3C), *Dimorphococcus* sp. excelled with the highest value, 28% DW, followed by *S. pantanale* (25% DW), and *W. botryoides*, *T. brasiliensis*, and *S. leptocladum* had approximately 20% DW. The other species had total lipids content lower than 15%, recalling here that such values were obtained at the end of the exponential growth phase. The values of biomolecules in mg.L^− 1^ are shown table S1 (supplementary material).

### Fatty Acid Profile

**Fig. 4:**
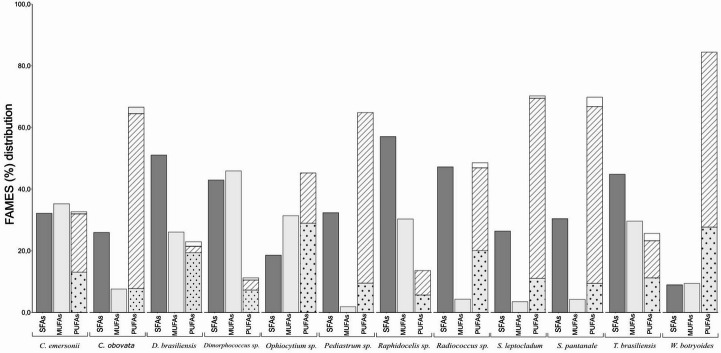
Distribution of fatty acid classes of the microalgae. SFAs (dark gray), MUFAs (light gray), and PUFAs, in which the presence of ω6 is represented as dots, ω3 as stripes, and other PUFAs as plain white. Values represent the means (*n* = 3)

The fatty acid composition is reported in Table 3. It shows the relative percentages of the main fatty acids identified. In complementation, Fig. 4 summarizes the total content of saturated (SFA), monounsaturated (MUFA), and polyunsaturated fatty acids (PUFA), as well as the proportions of ω3 and ω6 fatty acids and their corresponding ω6:ω3 ratio for the microalgae.

**Table 3:** Fatty acid profile of the microalgae. Values are presented as % of the total fatty acids and total content of Omegas 3 (ω3) and 6 (ω6), and Omega 6/3 ratio (ω6/3). Values represent the mean, and the standard deviations in parentheses (*n* = 3). Same letters represent means with no statistically significant differences (p-value < 0.05)

Microalgae	C16:0	C16:1 (n-7)	C18:0	C18:1 (n-9)	C18:1 (n-7)	C18:2 (n-6)	C18:3 (n-3)	C20:4 (n-6)	C20:5 (n-3)	C22:6 (n-3)	Others*	ω3	ω6	ω6/3
*C. emersonii*	27.71 (14.59)^cd^	0.67 (0.19)^c^	3.20 (0.09)^bcd^	34.56 (6.37)^a^	-	13.00 (0.36)^c^	-	-	-	-	17.75 (5.06)	18.93 (6.35)	13.04 (0.24)	0.81 (0.41)^b^
*C. obovata*	22.01 (4.52)^d^	7.46 (4.33)^b^	2.54 (0.01)^cde^	-	-	7.49 (1.30)^de^	46.81 (4.66)^b^	-	0.46 (0.05)^b^	-	12.22 (1.98)	56.74 (3.67)	7.52 (0.70)	0.14 (0.01)^b^
*D. brasiliensis*	47.87 (5.22)^a^	0.25 (0.44)^c^	3.14 (0.28)^bcd^	-	24.61 (2.47)^b^	19.18 (1.96)^b^	-	-	-	-	3.87 (1.13)	2.06 (0.51)	19.36 (1.41)	10.00 (2.65)^a^
*Dimorphococcus* sp.	40.86 (1.44)^abc^	-	2.05 (0.20)^cde^	-	45.88 (0.65)^a^	7.29 (0.21)^de^	-	-	-	-	0.48 (0.18)	3.26 (0.26)	6.91 (0.12)	2.23 (0.21)^b^
*Ophiocytium* sp.	12.48 (0.35)^a^	22.23 (3.69)^a^	6.06 (0.52)^a^	9.14 (6.89)^b^	-	6.33 (0.44)^de^	-	15.21 (0.23)^a^	12.7 (0.28)^a^	1.59 (0.27)^a^	10.53 (1.26)	16.31 (0.80)	28.90 (1.08)	1.77 (0.06)^b^
*Pediastrum* sp.	28.82 (3.75)^bcd^	-	2.09 (1.65)^cde^	-	1.05 (0.27)^cd^	8.82 (0.97)^d^	42.37 (2.75)^b^	-	-	-	14.96 (1.48)	55.35 (2.48)	9.36 (0.50)	0.17 (0.02)^b^
*Radiococcus* sp.	45.11 (2.02)^ab^	0.41 (0.36)^c^	1.82 (0.23)^de^	-	3.44 (0.69)^c^	20.14 (0.57)^b^	15.08 (0.79)^e^	-	-	-	10.31 (1.55)	26.83 (2.23)	20.05 (0.46)	0.76 (0.16)^b^
*Raphidocelis sp.*	48.73 (4.47)^a^	3.15 (0.05)^bc^	4.38 (0.71)^ab^	-	24.81 (2.04)^b^	5.32 (0.7)^e^	-	-	-	-	10.57 (1.28)	7.93 (2.09)	5.62 (0.52)	0.76 (0.08)^b^
*S. leptocladum*	24.88 (0.62)^cd^	3.44 (0.11)^bc^	1.21 (0.05)^e^	-	-	8.17 (0.58)^d^	33.13 (0.54)^c^	0.83 (0.07)^b^	0.12 (0.03)^c^	-	18.84 (0.14)	58.41 (1.05)	11.03 (0.48)	0.19 (0.02)^b^
*S. pantanale*	28.72 (3.15)^cd^	1.33 (0.73)^c^	1.66 (0.49)^de^	-	-	8.55 (1.18)^d^	33.08 (4.36)^c^	-	-	-	19.32 (2.35)	57.33 (1.55)	9.43 (0.35)	0.16 (0.01)^b^
*T. brasiliensis*	39.15 (0.24)^abc^	2.42 (0.15)^bc^	3.66 (0.15)^bc^	-	27.18 (0.02)^b^	11.99 (0.24)^c^	-	-	-	-	13.78 (0.17)	12.05 (0.01)	11.19 (0.24)	0.93 (0.32)^b^
*W. botryoides*	4.42 (7.66)^e^	5.73 (3.93)^bc^	4.50 (0.01)^ab^	-	3.63 (0.44)^c^	27.09 (1.04)^a^	54.62 (3.63)^a^	-	-	-	0 (0)	56.75 (0.58)	27.64 (0.41)	0.49 (0.00)^b^

*The complete table is available in supplementary material (Table S2).

The lipid profile of the 12 microalgae (Table 3) included 22 identified fatty acids, with 10 of them being predominant in most species, mainly PUFAs. Five microalgae had contents higher than 60% PUFAs: *C. obovata*, *Pediastrum sp.*, *S. leptocladum*, *S. pantanale*, and *W. botryoides* (Fig. 4). Alpha-linolenic acid (C18:3n-3) was the predominant component, followed by linoleic acid (C18:2n-6) (Table 3). Less common FAMEs for freshwater microalgae are eicosapentaenoic (EPA), docosahexaenoic (DHA), and arachidonic (ARA) acids, and although some of the algae presented these PUFAs, only in *Ophiocytium* sp. EPA (C20:5n-3), DHA (C22:6n-3), and ARA (C20:4n-6) were detected at concentrations higher than 1%. This microalga presented an ARA content of 15%, 1.3% for DHA, and 13% for EPA.

Regarding MUFAs, *Dimorphococcus* sp. had the highest content, mainly consisting of cis-vaccenic acid (C18:1n-7). *Ophiocytium* sp. had the second highest content, rich in palmitoleic acid (C16:1n-7). *C. emersonii* stood out in relation to oleic acid (C18:1n-9), comprising 34% of its total lipids. In this alga, MUFAs and SFAs were present in similar quantities, comprising each one about 35% of the total lipids, with palmitic acid (C16:0) as predominant SFA. Palmitic acid was also the main SFA in *Raphidocelis* sp. (60% of total lipids) and *D. brasiliensis* (50% of total lipids).

Among the ω3 and ω6 fatty acids, the two Charophyta species (*S. leptocladum* and *S. pantanale*) exhibited the highest ω3 levels, with ω3 being predominant over ω6. This favorable concentration of ω3 over ω6 was responsible for decreasing the ω6:3 ratio, which ranged from 0.1 to 2 in the microalgae investigated. The only exception was *D. brasiliensis*, with ω6:3 ratio of 10.0 ± 2.6.

### Pigments and Antioxidants

The concentration of the photosynthetic pigments, chlorophyll a, chlorophyll (Chl b, c_1_ and c_2_), and carotenoids, the antioxidant activity, and total polyphenols are shown in Table 4.

**Table 4:** Pigment composition of the microalgae: chlorophyll a (Chl a), chlorophyll (Chl b, c_1,_ c_2_), carotenoids (TC), the ratio of chlorophyll a to b (chl a/b), total polyphenols content (TP) and scavenging activity by DPPH (DPPH) in the 12 freshwater microalgae investigated. The values represent the means of three experimental replicates, and the standard deviation is shown in parentheses. Same letters represent means that did not show statistically significant differences among the microalgae (p-value < 0.05)

Microalgae	Chl a (mg.g^− 1^)	Chl b, c_1&2_* (mg.g^− 1^)	TC (mg.g^− 1^)	Chl a/b	TP (mgEq.g^− 1^)	DPPH (%_inhibition_)
*C. emersonii*	22.08 (0.89)^a^	11.05 (0.04)^a^	6.50 (0.35)^a^	2.00 (0.06)^abc^	7.03 (0.17)^bcd^	28.70 (1.15)^bcd^
*C. obovata*	14.60 (1.45)^bc^	10.98 (0.32)	—	—	11.01 (0.37)^a^	23.14 (3.95)^cde^
*D. brasiliensis*	7.49 (2.18)^d^	5.31 (0.47)^d^	1.90 (0.6)^e^	1.44 (0.28)^d^	6.85 (0.19)^bcd^	19.41 (2.31)^de^
*Dimorphococcus* sp.	12.16 (0.25)^cd^	8.47 (0.69)^bc^	2.85 (0.36)^cde^	1.44 (0.04)^d^	7.63 (0.25)^bc^	18.73 (0.87)^e^
*Ophiocytium* sp.	7.43 (1.64)^d^	6.40 (0.22)	—	—	5.88 (0.16)^d^	25.03 (4.90)^cde^
*Pediastrum* sp.	19.27 (3.44)^ab^	10.67 (0.96)^ab^	5.48 (0.58)^ab^	1.80 (0.07)^bcd^	8.07 (0.43)^b^	36.31 (2.27)^ab^
*Raphidocelis sp.*	8.36 (0.84)^d^	5.11 (0.62)^d^	2.28 (0.7)d^e^	1.64 (0.05)^bcdef^	6.47 (0.07)^bc^	20.88 (2.03)^bc^
*Radiococcus* sp.	12.71 (2.31)^cd^	8.65 (1.21)^abc^	3.61 (0.76)^cd^	1.46 (0.03)^d^	7.85 (0.08)^cd^	31.22 (2.62)^de^
*S. leptocladum*	15.19 (1.56)^bc^	6.53 (1.84)^cd^	4.34 (0.25)^bc^	2.33 (0.02)^a^	10.54 (0.18)^a^	42.68 (1.04)^a^
*S. pantanale*	10.04 (3.05)^cd^	5.95 (0.42)^d^	3.88 (0.08)^c^	1.69 (0.09)^bcd^	7.39 (0.34)^bc^	33.99 (2.67)^b^
*T. brasiliensis*	14.00 (0.42)^bc^	6.57 (0.26)^cd^	3.95 (0.77)^c^	2.13 (0.15)^ab^	6.69 (0.94)^bcd^	27.66 (1.61)^bcde^
*W. botryoides*	9.83 (0.78)^cd^	4.99 (0.41)^d^	3.20 (0.10)^cde^	1.96 (0.14)^abc^	3.43 (0.03)^e^	27.47 (0.88)^bcde^

* *C. obovata* is a Cryptophyceae and has chlorophyll c_2_ and *Ophiocytium* sp. is a Xanthophyceae and has chlorophyll c_1 and 2_.

In terms of photosynthetic pigments, the Chlorophyta and Charophyta followed the same pattern, with higher concentration of Chl a followed by Chl b. The highest Chl a were observed in *C. emersonii* (22.08 mg·g⁻¹ DW), *Pediastrum sp.* (19.27 mg·g⁻¹ DW), and *S. leptocladum* (15.19 mg·g⁻¹ DW), which also exhibited the highest Chl b content. Regarding total carotenoids (TC), *C. emersonii* (6.50 mg·g⁻¹), *Pediastrum sp.* (5.48 mg·g⁻¹), and *S. leptocladum* (4.34 mg·g⁻¹) presented the highest values, reinforcing their potential for pigment production. In contrast, *D. brasiliensis* and *Ophiocytium sp.* had the lowest Chl a concentration, lower than 7.5 mg·g⁻¹ DW, while *D. brasiliensis* and *Raphidocelis sp.* exhibited the lowest TC level, around 2.0 mg·g⁻¹ DW. The Chl a/b ratio was highest in *S. leptocladum* (2.33), being followed by *T. brasiliensis* (2.13), *C. emersonii* (2.00), and *W. botryoides* (1.96). Chl c was present in similar levels in *C. obovata* and *Ophiocytium* sp.

Considering the antioxidant potential, *S. leptocladum* showed 42.68% DPPH radical inhibition, followed by *Pediastrum* sp. with 36.31% inhibition. The total polyphenols (TP) content was highest in *C. obovata* (11.01 mgEq·g⁻¹) and *S. leptocladum* (10.54 mgEq·g⁻¹). However, despite its high TP content, *C. obovata* exhibited lower antioxidant activity (23.14% DPPH inhibition). The other microalgae presented TP values ranging from 3.43 to 8.07 mgEq·g⁻¹, with variable antioxidant activity.

### Principal Component Analysis

**Fig. 5:**
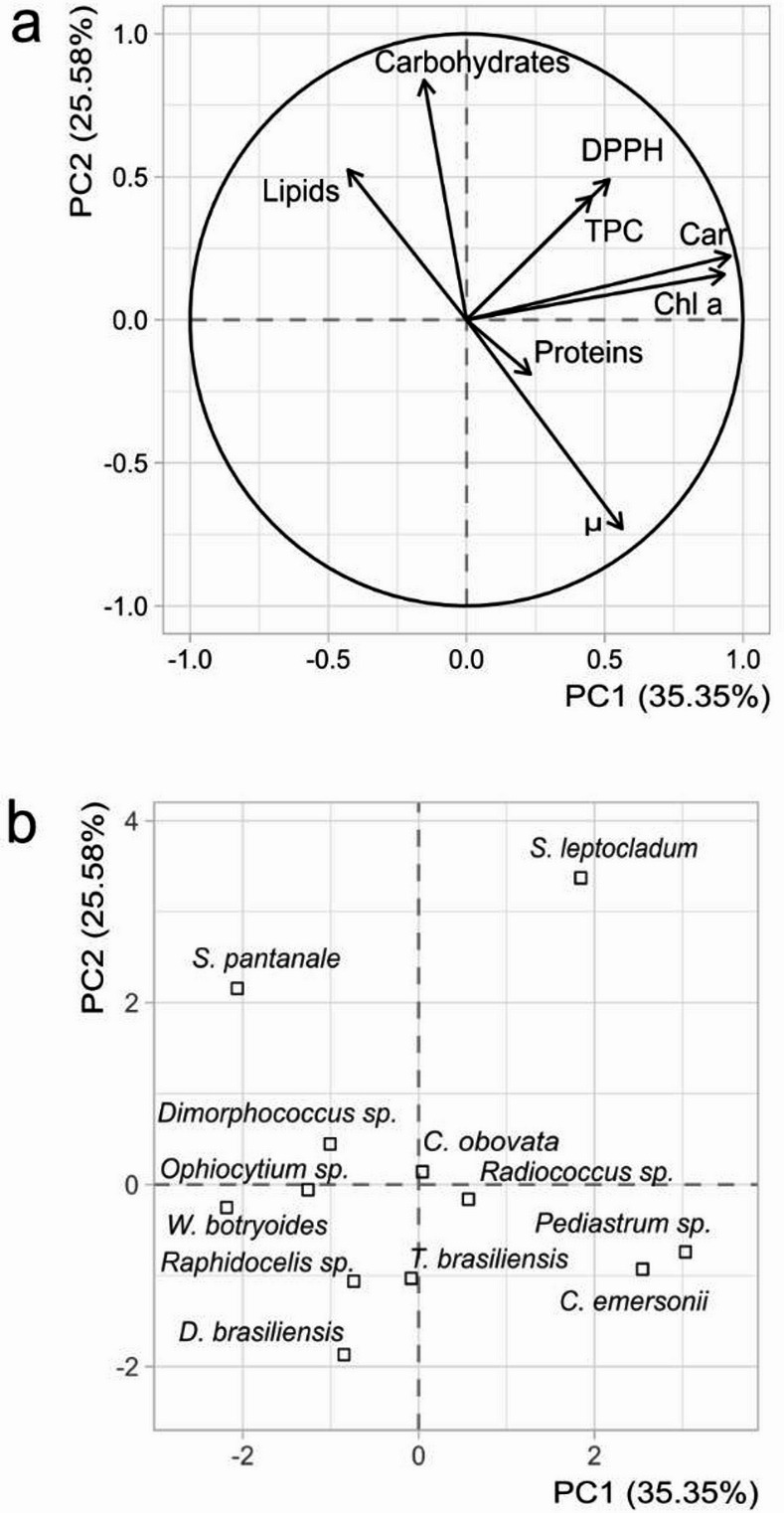
Principal component analysis of key biomolecules produced by the microalgae: total lipids, proteins and carbohydrates, chlorophyll a (Chl a), carotenoids (Car), total phenolic content (TPC), % inhibition DPPH radical (DPPH), and growth rate (µ), considering (**a**) variables and (**b**) individuals. The individual plot represents the mean of three experimental replicates (*n* = 3)

Considering growth rate, biomolecules (proteins, carbohydrates, and lipids), pigments (Chl a and carotenoids), photosynthetic parameters and antioxidant compounds, a principal component analysis (PCA) plot was generated (Fig. 5). For *C. obovata* and *Ophiocytium* sp. the values of carotenoids and Chlorophyll c were not considered using the R-package missMDA to correct the missing values [27]. The loading values for PC1 and PC2 are present as table S3 in supplementary material.

In the PCA of the observed variables (Fig. 5a), PC1 explained 35.35% of the total variance and was positively correlated with most variables, except carbohydrates, lipids, and polyphenols. PC2 accounted for 25.58% of the variance, showing positive correlations with lipids and carbohydrates. Together, these two components explained 60% of the variation in the dataset. Some patterns can be mentioned, including a correlation between growth rate and protein content, although it is not a strong correlation. Direct and strong correlations were obtained for the antioxidant activity (DPPH) with the pigments chlorophyll a, and carotenoids, and antioxidant activity with total polyphenols content.

The PCA individuals plot (Fig. 5b) revealed that PC1 was associated with algae exhibiting the highest protein content and pigment production, such as *C. emersonii* and *Pediastrum* sp., whereas *S. leptocladum* was notably linked to antioxidant activity and elevated levels of polyphenol. PC2 highlighted the accumulation of lipids and carbohydrates, with *Staurastrum leptocladum*,* Staurastrum pantanale*, and *Dimorphococcus* sp. standing out for their capacity to produce these molecules.

## Discussion

### Growth Rates and Biomass

The variation of growth rates among the microalgae confirms that this is a species-specific variable [28], particularly with growth conditions such as nutrients in excess and light supplied in the respective species Ek. Although smaller cells tend to have higher growth rates, *Pediastrum* sp., which has cellular size ranging from 5 to 30 μm and coenobium from 50 to 200 μm, depending on the species [29] had growth rate (1.0 d^− 1^) similar to *Chlorella emersonii* (0.9 d^− 1^), whose cell size is around 10 μm [30]. *S. leptocladum* and *S. pantanale* had the lowest growth rates among the organisms investigated, which can be related to their cell size [31] that may reach values as high as 85–97 μm for *S. leptocladum* [32] and 37–43 μm for *S. pantanale* [33].

The lack of correlation between growth rate and biomass confirms other literature results. This indicates that biomass generation is influenced by other variables in addition to growth rate. For example, *S. leptocladum* cultures had the highest biomass (190 mg.L^− 1^), but the lowest growth rate (0.2 d^− 1^). *S. leptocladum* can reach a size of up to 47 μm [34], being a large species. The more than double biomass present in *S. leptocladum* cultures in comparison to *S. pantanale* can be related to the nearly double the cell size of the first [32, 33].

### Rapid Light Curve

The RLC, can be considered a measure of photosynthetic rate that is related to irradiance in a non-linear fashion. It permitted the detection of differences in the overall photosynthetic performance of the microalgae. RLC reflects the short-term light history of the algae, therefore indicating the actual state of photosynthesis [35]. Considering that the microalgae were grown under nutrient replete conditions and at light intensity representative of its specific E_k_, the different RLC profiles obtained can be related to the intrinsic properties of the organisms. The results of low α obtained for the *S. leptocladum* and *S. pantanale* (Charophyta) in the present research are in accordance with those in Stamenković et al. [36], one of the few investigations that analyzed RLC in different species of Charophyta, all belonging to the *Cosmarium* genus isolated from different geographical distribution. The authors showed that arctic and tropical *Cosmarium* strains were adapted to high light, even though with low α, e.g., around 0.2, similar to the present results. Different from our study that used light intensity close to the specific E_k_ to grow the organisms, Stamenković et al. [31] kept the microalgae under low light, 30 µmol photons.m^− 2^.s^− 1^. Different from the present study, the E_k_ values they obtained varied within 485–830 µmol photons.m^− 2^.s^− 1^ depending on the strain. This means the authors obtained E_k_ values 2 to 4 times higher than those obtained in the present study for the Charophyta. Therefore, the tropical Charophyta *S. leptocladum* and *S. pantanale* (this study), were photosynthetically more efficient under moderate light if compared to Stamenković et al. [36] results. The authors considered their species to be adapted to high solar radiation independent of its geographical origin. In the present study, the rETRmax and α were supportive to the low growth rate obtained for the Charophyta species.

The Chlorophyta *Pediastrum* sp. and *C. emersonii* stood out in relation to rETRmax, as well as growth rate and biomass productivity. Alpha was highest in *Pediastrum* sp., but apart from that most other species had α with no statistically significant differences and within expected values. Schwaderer et al. [37] compared different taxonomic groups of microalgae and showed that the Chlorophyta were adapted to high light intensities, showing higher growth rates and lower α than other groups. Differently, our results showed that the Chlorophyta (8 species) had the highest α values comparing to microalgae belonging to Charophyta (*S. leptocladum* and *S. pantanale*), Cryptista (*Cryptomonas obovata*) and Heterokontophyta (*Ophiocytium* sp.).

According to Sakshaug et al. [38], several factors can contribute to low values of the maximum light utilization coefficient (α ). Situations where environmental conditions induce to cyclic electron flow, photorespiration, loss of functional reaction centers, activity of xanthophyll cycle and/or packaging of pigments inside the cells, which may reduce the absorption efficiency of the pigments are physiological processes that can impact on the α value. Such situations may result from variations in light intensity, temperature and nutrients availability in the culture flask. Because we used batch cultures and therefore population accumulates inside and consume nutrients, even though cultures were sampled in exponential growth, self-shading may have occurred. Moreover, considering that *S. leptocladum* and *S. pantanale* are large organisms, they may feel such effects more than the smaller ones, therefore showing the lowest α among the 12 strains. From the present results we conclude that although several factors can influence the efficiency with which the cells use incident light and affect the rate of electron transport, these variables can also be species-specific.

### Biochemical Composition

In the present research, *C. emersonii*, *Radiococcus* sp., *Dimorphococcus* sp., and *Pediastrum* sp., with total proteins content ~ 60% DW, stood out in comparison to the other Chlorophyta and those reported in literature. Ma et al. [39] obtained up to 54% proteins in *Chlorella sorokiniana* in 1 L batch cultures, and Silva et al. [40] obtained 32% for *Chlorella fusca* in exponential growth phase of 250 L culture kept in a greenhouse. In contrast to the present results, Illman et al. [41] obtained 32% total proteins in stationary phase cells of *Chlorella emersonii*. The protein values reported in both Silva et al. [40] and Illman et al. [41] ​​were half of those obtained in the present work. The protein content can be species specific, but comparing to large scale cultures, in which environmental conditions vary daily, the higher protein content of *C. emersonii* in the present study can be a consequence of a more stable and controlled environment. The lower protein content obtained by Illman et al. [41] can be associated with harvesting the cells in stationary growth phase cultures. It is known that cultures in the exponential growth phase contain higher protein levels than those in stationary phase, as microalgae in the stationary phase may experience degradation of chloroplast proteins [42]. The positive correlation between total proteins and growth rates obtained in the PCA analysis in this study confirms that higher growth rates are associated with increased total proteins content, what is in accordance with the results of Finkel et al. [43]. Using a hierarchical Bayesian analysis of data compiled from literature, the authors showed that the median macromolecular composition of microalgae as percent dry weight in exponential growth was 32.2% proteins, 17.3% lipids, 15.0% carbohydrates, 17.3% ash, and a small part as chlorophyll-a (1.1%). The positive correlation we observed between growth rate and proteins content in microalgae is likely to be due to the substantial protein synthesis and expression that occur at high cell division rates, as demonstrated by Rees et al. [44]. The authors observed a correlation between growth rate and increased RNA content in photosynthetic eukaryotes. Based in the above information, we suggest that if proteins are the final product of interest, microalgae cultures should be harvested in exponential growth phase.

Microalgae carbohydrates have been proposed as sustainable source of prebiotics, offering health functionality [45] and showing potential for use in bioenergy [46]. In this research, the genus *Staurastrum*, (*S. pantanale* and *S. leptocladum*) presented the highest carbohydrates content in comparison to the other species. This genus is known for its dense carbohydrate sheath surrounding the cells [47], therefore the higher values can have the contribution of exopolysaccharides. The inverse correlation between carbohydrates and proteins we obtained in the PCA analysis supports the results in Reitan et al. [48], who noted that conditions favoring lower growth rates would lead to increased carbohydrates and lipids in microalgae. However, different from their study that focused on the effects of temperature and N/C ratios on growth rates and biochemical composition of several microalgae, in our study, no modification in culture medium or growing conditions were performed. Therefore, the growth rates we obtained reflect intrinsic properties of exponentially growing cells of the different microalgae. We observe that physiological studies examining biomolecule composition in this algal group remain scarce in the literature.

In the present study, the production of total lipids was inversely correlated to growth rates in the PCA analysis. Stamenković et al. [49] reported that *Staurastrum* spp. (Charophyta) have the potential for lipids accumulation. Consistent with their findings, the present study showed lipid content of 25% DW in *S. pantanale* and 30% in *Dimorphococcus* sp., indicating that both can be considered high lipid producers. This is noteworthy given that the cultures were harvested in exponential growth phase yet exhibited such high lipid content.

Overall, the biomolecules content observed in the microalgae in this study reflect the growth conditions, timing of culture harvesting and species-specific characteristics. The generally high values of total proteins can be related to the high nitrogen concentration in BG11 (1.5 g·L⁻¹) culture medium and the fact that biomolecules were determined in exponentially growing cells. Nitrogen is a macronutrient required for microalgae growth, being a key factor for cell division, nucleic acids, enzymes and other cellular proteins [50], therefore affecting its biochemical composition. Literature has shown that when cells are nitrogen depleted, cell division decreases, and the cells accumulate lipids in addition to affecting the quality of such biomolecule by increasing their saturation [51]. Carbohydrates and lipids, which varied among species and can be related to species-specific traits in addition to the culturing conditions.

### Fatty Acids Profile

In this research, the Charophyta (~ 60%) and Chlorophyta (~ 50%, with *W. botryoides* reaching 80%) were the phyla with higher PUFA content. The Chlorophyta microalgae are well known for their richness in ω3 fatty acids (Guedes et al. [52], Matos et al. [53], Santhakumaran et al. [54]), but less information is available on the fatty acid composition of Charophyta microalgae. A recent publication, Rahaman et al. [55], which investigated the fatty acid composition of 9 filamentous Charophyta (8 Zygnematophyceae and 1 Klebsormidiophyceae) showed that most of them had from 50 to 60% PUFA, less than 10% MUFA except for a *Zygnema* sp. strain (~ 70% MUFA), and ~ 30% SFA. In general, the values obtained in the present research for the two single cell Charophyta, *S. leptocladum* and *S. pantanale*, are consistent with those of Rahman et al. [55] that investigated the filamentous ones.

Guedes et al. [52] investigated microalgae of the taxonomic Class Chlorophyceae, Bacillariophyceae, Prymnesiophyceae, Eustigmatophyceae and Rhodophyceae, and concluded that the Chlorophyceae accumulated higher PUFA than the other Classes. Matos et al. [53] observed that *Chlorella vulgaris* had 40% PUFAs in relation to the total fatty acids, higher than the other groups they evaluated, which were mostly below 2%. Evaluating the fatty acid profile of 14 Chlorophyta, Santhakumaran et al. [54] obtained that a microalgae strain belonging to the Scenedesmaceae taxonomic family, the same as *W. botryoides* (this research) had PUFA levels exceeding 50%.

The interest in ω3 fatty acid is related to its essentiality and impact on the maintenance of healthy physiological functions and anti-inflammatory properties [56]. In this research, one strain of Chlorophyta, *W. botryoides*, stood out for its PUFA content in comparison to the other species. It was the only one with over 80% of its total fatty acids as PUFAs, of which 50% were ω3. These results are in accordance with Castro et al. [57] that reported 55% of alpha-linolenic (ω3) and 27% linoleic (ω6) for *W. botryoides*. In the present research, the second microalgae in the rank with high PUFA content was *Pediastrum* sp. (65%), being attributed to linolenic acid, which comprised nearly half of its total fatty acids. This leads to a favorable ω6:ω3 ratio of 0.17 in *Pediastrum*. The ratio between these groups of fatty acids is important from a nutritional point of view, as ideally, it is recommended an intake of 4ω3:1ω6. Diets with higher proportions of ω6 fatty acids influence the onset of inflammation-related diseases and chronic illnesses [58], whereas the opposite, diets rich in ω3, have anti-inflammatory properties. This makes *Pediastrum* sp. an interesting organism for future studies related to nutritional applications, as it is an alga that produces large amounts of antioxidants and PUFAs, particularly as a vegan source of ω3. This would be of interest for human and animal nutrition [59–61]. *Ophiocytium* sp. was the only microalga among the 12 investigated in this study that produced ARA, EPA, and DHA. This may be characteristic of the Xanthophyceae class (phylum Heterokontophyta), to which *Ophiocytium* sp. belongs. Lang et al. [62] evaluated the lipid composition of over 2,000 microalgae strains belonging to various groups and observed that, among the Xanthophyceae, almost half of them had arachidonic acid (ARA), against just 14% of other taxonomic classes belonging to the Chlorophyta phylum.

The other microalgae species we studied showed more pronounced levels of MUFAs and SFAs compared with the species mentioned above. According to literature, the saturated fatty acids, such as palmitic acid and monounsaturated acids such as the oleic acid are desirable for bioenergy [63, 64]. For such application, it is also recommended that PUFA values do not exceed 17% [65]. This composition directly affects the quality features of biodiesel, with the cetane number (CN) being one of the main indicators [66]. Higher proportions of C16:0 and C18:0 tend to increase CN values, whereas the presence of C18:3 is associated with lower-quality oil [66, 67]. However, high levels of saturated fatty acids (SFAs) lead to higher freezing points and reduced lubricity. Therefore, the presence of monounsaturated fatty acids (MUFAs) is essential to improve the cold-flow properties and lubricity of biodiesel without compromising ignition quality [68, 69]. Considering these premises, the microalgae *Dimorphococcus* sp. was a promising strain for the bioenergy sector. In exponentially growing cells, *Dimorphococcus* sp. had the highest production of lipids (28%), with almost 50% MUFAs and 41% SFAs, in addition to high protein levels as high as 56% DW. We highlight that lipids and proteins were antagonistic in the PCA, thus *Dimorphococcus* sp. can be considered an exception among the strains. Its growth rate also showed an inverse relationship with lipid content, where carbohydrates were the only factor that correlated with higher lipid percentages. This may be related to the fact that both play a fundamental role in various structural processes and act as carbon storage [70].

### Pigments and Antioxidants

The pigments, chlorophylls a (Chl a) and b (Chl b), and total carotenoids produced by the 12 strains studied followed the pattern reported in Azaman et al. [71], in which the Chlorophyta *Chlorella sorokiniana* and *Chlorella zofingiensis* presented higher content of Chl a, followed by Chl b and carotenoids. In the present results, the PCA analysis confirmed a positive correlation between Chl a and carotenoids, and Chl a and Chl b. According to Larkum [72] this is an expected pattern in exponentially growing photoautotrophic microalgae, as Chl a is the main photosynthetic pigment in photosystems I and II. Usually, there are around 7 or 8 Chl a to 5 Chl b per photosystem, resulting in a Chl a/b ratio near 1.4 [73]. This is related to the optical cross section of the light harvesting antenna and photosynthetic efficiency. By manipulating the content of chlorophyll b, the photosynthetic efficiency may be improved, therefore a strategy to increase production in photosynthetic organisms. According to Negi et al. [74], this can be achieved by reducing chlorophyll b levels. In plants, Friedland et al. [75] identified a sharp transition point in pigment concentrations and antenna size, where deviations from the optimum, either by increasing or decreasing the antenna size, were associated with lower photosynthetic efficiency. Therefore, an ideal antenna size can lead to increased productivity in microalgae. The ratios we detected (1.44–2.33) indicate physiological health of the photosystems and, according to Negi et al. [74] higher biomass productivity for the microalgae with higher Chl a/Chl b ratio can be expected. Although the PCA did not show a direct correlation between Chl a/Chl b ratio and productivity for the organisms altogether, the organisms that had the highest Chl a/Chl b ratio, also had the highest biomass productivity. This applies to the Charophyta *S. leptocladum* and the Chlorophyta *Pediastrum* sp. and *Chlorella emersonii*. According to Cogne et al. [76] and Friedland et al. [75], higher Chl a content relative to Chl b can be associated with better photosynthetic performance, greater biomass accumulation, and higher growth rates in microalgae and plants.

Carotenoids, although in smaller amounts when compared to the chlorophylls, play a complementary role in light absorption and act as free radical inhibitors [77]. In this study, a correlation was observed between total carotenoid content and antioxidant response. However, this correlation was influenced by the polyphenol content in the cells, which affects DPPH scavenging activity due to its natural ability to stabilize free radicals, either by donating protons or electrons [78]. This is confirmed by the PCA analysis that showed a correlation between increased antioxidant potential, carotenoids and polyphenol content for the 12 microalgae studied. The main contributor to such result was the microalga *S. leptocladum*, which presented the highest antioxidant activity and total polyphenols compared to the other microalgae. Another alga with important antioxidant potential was *Pediastrum* sp. (36%), which also showed significant inhibition of the DPPH radical. These results are in partial agreement with those in Corrêa da Silva et al. [79]. The authors investigated the microalga *Pediastrum* sp. and achieved 39.8% inhibition of the ABTS radical, demonstrating its capacity to produce antioxidant compounds. However, they obtained four times lower polyphenol concentration (1.95 mgEq.g^− 1^) compared to that in the present study for the same genus (8.07 mgEq.g^− 1^). Santiago-Díaz et al. [72] obtained DPPH radical inhibition values of up to 30% in methanolic extracts of three Chlorophyta microalgae, similar to the results for the microalgae examined in the present study. Banskota et al. [81] observed up to 45% DPPH radical inhibition activity in *Tetraselmis chuii* and around 21% in *Chlorella sorokiniana*. For total polyphenols, the authors obtained 9.78 and 6.32 mgEq.g^− 1^, comparable to most of the microalgae assessed in the present study. Using various solvent mixtures and extraction methods, Monteiro et al. [82] achieved a maximum of 6 mgEq.g^− 1^ in different groups of macro and microalgae. In our research, only *Ophiocytium* sp. and *W. botryoides* presented polyphenol content lower than 6 mgEq.g^− 1^.

In the present study, no clear correlation or species-specific effect was observed between the different groups of microalgae and their antioxidant capacity. According to Koletti et al. [83], antioxidants in microalgae play a key role in inhibiting free radicals generated during natural processes such as photosynthesis. These compounds act synergistically, and although pigments and polyphenols contribute to the antioxidant response, they represent only part of the complex mechanisms involved [77]. Nevertheless, our results showed that some species (*Ophiocytium* sp. and *W. botryoides*) may be promising candidates for future studies aiming at enhancing antioxidant production for commercial applications.

## Conclusion

The present study prospected 12 microalgae belonging to different taxonomic groups and highlighted promising organisms for specific applications. *C. emersonii* and *Pediastrum* sp. stood out due to their high growth rates and production of biomass, proteins, and pigments. Additionally, *Pediastrum* sp. exhibited one of the highest antioxidant activities and produced essential polyunsaturated fatty acids in higher quantities than *C. emersonii*. Therefore, due to the combination of its antioxidant potential, polyunsaturated fatty acid content, and high protein levels, *Pediastrum* sp. is suggested as a promising organism for nutritional applications. *Dimorphococcus* sp., emerged as an important protein producer, with additional potential for application in the bioenergy sector. This is associated with its high lipid content, superior to that of the other species evaluated, in addition to its fatty acid composition. Presenting high MUFA content, which supports desirable properties for biodiesel, *Dimorphococcus* sp. is suggested as an organism for further research on the optimization of biofuels from microalgae. Microalgae of the genus *Staurastrum* (*S. leptocladum* and *S. pantanale*), and *W. botryoides* were rich in carbohydrates and essential polyunsaturated fatty acids, while *Ophiocytium* sp. excels in the production of uncommon essential fatty acids, such as DHA, EPA, and ARA.

The results of this study demonstrate the potential of microalgae from understudied taxonomic groups as sources of industrially relevant biomolecules. The unique and advantageous characteristics of these microalgae make them promising candidates for the development of novel products and processes in various biotechnology fields within a circular economy framework.

## Supplementary Information

Below is the link to the electronic supplementary material.

ESM1 (DOCX.34.2 KB)

## Data Availability

Data will be made available upon request.
