# Supplementary material for: Assessing the Physiology and Biochemistry of Freshwater Microalgae for Biotechnological Applications
Source: Appl Biochem Biotechnol. 2026 Feb 2;198(4):2806–30. doi: 10.1007/s12010-025-05578-6 (PMC13033007; doi:10.1007/s12010-025-05578-6)
Supplement: Supplementary file 1 — (DOCX.34.2 KB) [file 12010_2025_5578_MOESM1_ESM.docx]

**Biochemical and physiological characterization of 12 freshwater microalgae reveals Pediastrum sp. as a promising species for nutrition and Dimorphococcus sp. for biofuels**

| Table S1. Biochemical composition of microalgae (proteins, carbohydrates, and lipids reported in mg L⁻¹). Values represent the mean with the standard deviation of the mean shown in parentheses (n=3). | | | |
| --- | --- | --- | --- |
| Microalgae | Proteins | Carbohydrates | Lipids |
| *C. emersonii* | 80.47 (6.73) | 11.35 (1.84) | 10.08 (2.08) |
| *C. obovatum* | 23.1 (4.85) | 7.86 (1.85) | 7.15 (0.2) |
| *D. brasiliensis* | 47.52 (9.78) | 8.5 (6.90) | 11.4 (3.6) |
| *Dimorphococcus sp.* | 69.96 (0.00) | 18.26 (1.84) | 35.49 (4.91) |
| *Ophiocytium sp.* | 45 (3.97) | 15.19 (4.23) | 7.63 (0.27) |
| *Pediastrum sp.* | 80.02 (6.82) | 10.32 (1.90) | 18.5 (1.04) |
| *R. piracicabana* | 39.44 (7.57) | 8.86 (1.38) | 14.55 (3.65) |
| *Radiococcus sp.* | 93.67 (21.0) | 37.79 (0.46) | 7.63 (0.27) |
| *S. leptoclatum* | 75.55 (5.99) | 60.74 (9.95) | 33.85 (3.35) |
| *S. pantanale* | 25.61 (1.82) | 27.22 (6.16) | 16.35 (0.55) |
| *T. brasiliensis* | 29 (9.65) | 7.33 (1.17) | 16.81 (1.91) |
| *W. botryoides* | 19.62 (0.9) | 8.27 (1.55) | 11.68 (3.02) |

| Table S2. Complete fatty acid profile of the microalgae. Values are presented as % of the total fatty acids and total content. Values represent the mean, and the standard deviations in parentheses (n = 3). Same letters represent means with no statistically significant differences (p-value < 0.05). | | | | | | | | | | | | |  |
| --- | --- | --- | --- | --- | --- | --- | --- | --- | --- | --- | --- | --- | --- |
| FAMES | *C. emersonii* | *C. obovata* | *D. brasiliensis* | *Dimorphococcus* sp. | *Ophiocytium* sp*.* | *Pediastrum* sp*.* | *Radiococcus* sp*.* | *Raphidocelis sp.* | *S. leptocladum* | *S. pantanale* | *T. brasiliensis* | *W. botryoides* | |
| C16:0 | 27.71  (14.59)^cd^ | 22.01  (4.52)^d^ | 47.87  (5.22)^a^ | 40.86  (1.44)^abc^ | 12.48  (0.35)^a^ | 28.82  (3.75)^bcd^ | 45.11  (2.02)^ab^ | 48.73  (4.47)^a^ | 24.88  (0.62)^cd^ | 28.72  (3.15)^cd^ | 39.15  (0.24)^abc^ | 4.42  (7.66)^e^ | |
| C16:1(n-7) | 0.67  (0.19)^c^ | 7.46  (4.33)^b^ | 0.25  (0.44)^c^ | - | 22.23  (3.69)^a^ | - | 0.41  (0.36)^c^ | 3.15  (0.05)^bc^ | 3.44  (0.11)^bc^ | 1.33  (0.73)^c^ | 2.42  (0.15)^bc^ | 5.73  (3.93)^bc^ | |
| C16:1(n-9) | - | - | - | - | - | - | - | 1.5  (0.05)^a^ | - | 0.69  (0.64)^b^ | - | - | |
| C16:2(n-4) | - | - | - | - | 10.53  (1.89)^a^ | - | - | - | - | - | - | - | |
| C16:2(n-6) | - | 0.21  (0.22)^bc^ | - | 0.48  (0.24)^bc^ | - | 0.70  (0.17)^bc^ | 0.39  (0.34)^bc^ | - | 1.98  (0.17)^a^ | 0.91  (0.71)^b^ | 0.20  (0.06)^bc^ | - | |
| C16:3(n-3) | - | 1.15  (0.19)^ef^ | 0.58  (0.36)^ef^ | - | - | 3.31  (0.22)^d^ | 4.88  (0.05)^c^ | 1.25  (0.89)^ef^ | 15.76  (0.2)^a^ | 12.57  (1.38)^b^ | 1.82  (0.04)^e^ | - | |
| C16:4(n-3) | 16.35  (7.60)^a^ | 9.82  (2.55)^ab^ | 1.01  (1.10)^c^ | - | - | 8.68  (1.90)^b^ | 4.37  (1.70)^bc^ | 3.92  (1.37)^bc^ | - | - | 10.06  (0.00)^ab^ | - | |
| C18:0 | 3.20  (0.09)^bcd^ | 2.54  (0.01)^cde^ | 3.14  (0.28)^bcd^ | 2.05  (0.20)^cde^ | 6.06  (0.52)^a^ | 2.09  (1.65)^cde^ | 1.82  (0.23)^de^ | 4.38  (0.71)^ab^ | 1.21  (0.05)^e^ | 1.66  (0.49)^de^ | 3.66  (0.15)^bc^ | 4.50  (0.01)^ab^ | |
| C18:1(n-9) | 34.56  (6.37)^a^ | - | - | - | 9.14  (6.89)^b^ | - | - | - | - | - | - | - | |
| C18:1(n-7) | - | - | 24.61  (2.47)^b^ | 45.88  (0.65)^a^ | - | 1.05  (0.27)^cd^ | 3.44  (0.69)^c^ | 24.81  (2.04)^b^ | - | - | 27.18  (0.02)^b^ | 3.63  (0.44)^c^ | |

| Table S2cont. Complete fatty acid profile of the microalgae. Values are presented as % of the total fatty acids and total content. Values represent the mean, and the standard deviations in parentheses (n = 3). Same letters represent means with no statistically significant differences (p-value < 0.05). | | | | | | | | | | | | |
| --- | --- | --- | --- | --- | --- | --- | --- | --- | --- | --- | --- | --- |
| FAMES | *C. emersonii* | *C. obovata* | *D. brasiliensis* | *Dimorphococcus* sp*.* | *Ophiocytium* sp. | *Pediastrum* sp. | *Radiococcus* sp. | *Raphidocelis sp.* | *S.*  *leptocladum* | *S. pantanale* | *T. brasiliensis* | *W. botryoides* |
| C18:1(n-5) | - | - | 1.19  (0.30)^b^ | - | - | - | - | - | - | 2.2  (0.88)^a^ | - | - |
| C18:2(n-6) | 13.00  (0.36)^c^ | 7.49  (1.30)^de^ | 19.18  (1.96)^b^ | 7.29  (0.21)^de^ | 6.33  (0.44)^de^ | 8.82  (0.97)^d^ | 20.14  (0.57)^b^ | 5.32  (0.7)^e^ | 8.17  (0.58)^d^ | 8.55  (1.18)^d^ | 11.99  (0.24)^c^ | 27.09  (1.04)^a^ |
| C18:3(n-6) | - | - | 1.09  (0.3)^b^ | - | - | - | - | - | 0.93  (0.22)^b^ | 2.96  (0.47)^a^ | 0.3  (0.09)^c^ | - |
| C18:3(n-3) | - | 46.81  (4.66)^b^ | - | - | - | 42.37  (2.75)^b^ | 15.08  (0.79)^e^ | - | 33.13  (0.54)^c^ | 33.08  (4.36)^c^ | - | 54.62  (3.63)^a^ |
| C18:4(n-3) | 3.12  (1.27)^cd^ | 0.36  (0.20)^fg^ | 1.06  (0.28)^efg^ | 3.44  (0.4)^cd^ | - | 1.89  (0.29)^def^ | 3.67  (0.37)^c^ | 2.21  (0.55)^cde^ | 9.24  (0.57)^a^ | 7.35  (1.09)^b^ | 1.24  (0.08)^efg^ | - |
| C20:4(n-6) | - | - | - | - | 15.21  (0.23)^a^ | - | - | - | 0.83  (0.07)^b^ | - | - | - |
| C20:5(n-3) | - | 0.46  (0.05)^b^ | - | - | 12.7  (0.28)^a^ | - | - | - | 0.12  (0.03)^c^ | - | - | - |
| C22:0 | - | 0.81  (0.05)^b^ | - | - | - | 1.08  (0.26)^b^ | 0.24  (0.1)^c^ | 2.36  (0.38)^a^ | - | - | 0.86  (0.1)^b^ | - |
| C22:1 | - | - | - | - | - | - | 0.43(0.04)^a^ | - | - | - | - | - |
| C22:6(n-3) | - | - | - | - | 1.59  (0.27)^a^ | - | - | - | - | - | - | - |
| C24:0 | 1.21  (0.19)^b^ | 0.23  (0.06)^de^ | - | - | - | 0.34  (0.22)^cd^ | - | 1.54  (0.02)^a^ | 0.17  (0.02)^de^ | - | 0.55  (0.09)^c^ | - |
| C24:1 | - | - | - | - | - | 0.8(0.2)^a^ | - | - | - | - | - | - |

| Table S3. Loading values for the main principal components, PC1 (35.35%) and PC2(25.58%). Abbreviations: Chl a chlorophyll a, Car carotenoids, TPC total phenolic content, DPPH % inhibition DPPH radical, and growth rate (µ). | | | | |
| --- | --- | --- | --- | --- |
| Variable | Factor 1 | Factor 2 |  |  |
| µ | 0,566 | -0,727 |  |  |
| Proteins | 0,248 | -0,179 |  |  |
| Lipids | -0,423 | 0,527 |  |  |
| Carbohydrates | -0,152 | 0,839 |  |  |
| Chl a | 0,94 | 0,164 |  |  |
| Car | 0,944 | 0,228 |  |  |
| TPC | 0,413 | 0,418 |  |  |
| DPPH | 0,525 | 0,498 |  |  |
